# Dermatologische Versorgung von älteren Menschen mit Psoriasis vor und nach Eintritt in ein Pflegeheim: Eine qualitative Analyse aus Sicht von medizinischen Versorgern

**DOI:** 10.1007/s00105-022-04989-4

**Published:** 2022-04-28

**Authors:** C. C. von Stuelpnagel, J. Petersen, M. Augustin, R. Sommer

**Affiliations:** Institut für Versorgungsforschung in der Dermatologie und bei Pflegeberufen (IVDP), Universitätsklinikum Hamburg-Eppendorf (UKE), Martinistr. 52, 20246 Hamburg, Deutschland

**Keywords:** Fokusgruppen, Versorgungsbedarf, Pflege, Digitale Versorgung, Leitlinie, Dermatologie, Focus groups, Healthcare needs, Care, Remote consultations, Guideline, Dermatology

## Abstract

**Hintergrund:**

Demografische Veränderungen bewirken einen steilen Anstieg der Anzahl der über 65-Jährigen. Damit verbunden ist die Zunahme der Anzahl pflegebedürftiger, multimorbid Erkrankter. National wie auch international gibt es keine Informationen insbesondere zur Versorgung von Psoriasiserkrankten im Setting Pflegeheim und zur Frage, wie diese durch den Eintritt in ein Pflegeheim beeinflusst wird.

**Fragestellung:**

Ziel war es, anhand von Interviews bzw. Fokusgruppen die Ergebnisse vorausgehender Routinedatenanalysen zur Versorgung Psoriasiserkrankter in Pflegeheimen mit medizinischen Versorgern (Dermatologen, Allgemeinmediziner, Pflegedienstleitungen und Pflegekräfte) zu diskutieren, Schwierigkeiten der Versorgung aufzudecken und abschließend Handlungsempfehlungen für eine zukunftsfähige gerontodermatologische Versorgung abzuleiten.

**Material und Methoden:**

Durchgeführt wurden qualitative Leitfaden-gestützte Interviews und Fokusgruppen mit Dermatologen (*n* = 5), Allgemeinmedizinern (*n* = 7), Pflegekräften (*n* = 7) und Pflegedienstleitungen (*n* = 2). Die Daten wurden inhaltsanalytisch ausgewertet.

**Ergebnisse:**

Die Auswertung ergab insgesamt 344 Aussagen, die insgesamt 14 Hauptkategorien zugeordnet werden konnten. Die Ergebnisse zeigen, dass für die Versorgungsqualität von Menschen mit Hautkrankheiten, insbesondere Psoriasis, in Pflegeinrichtungen ein Verbesserungsbedarf besteht. Dieser zeigt sich sowohl auf ärztlicher als auch auf pflegerischer Ebene. Laut der Versorger (*N* = 21) kann dies insbesondere durch eine verstärkte digitale Versorgung, dermatologische Schulungen für Hausärzte und Pflegefachkräfte sowie engere Kooperationen zwischen den einzelnen Disziplinen adressiert werden.

**Schlussfolgerung:**

Digitale Pflegekonsile, aber auch eine spezifische Leitlinie zur „Haut des alternden Menschen“ können von Nutzen sein, um die dermatologische Versorgungssituation im Pflegeheim zu verbessern und somit das Wohlbefinden der Betroffenen zu steigern.

## Abstract (en)

**Background:**

Demographic changes are causing a rapid increase in the number of people over 65 years of age. This is associated with an increase in the number of multimorbid patients in need of care. Nationally as well as internationally, there is little information on the treatment of psoriasis patients in the nursing home setting and on the question of how this is influenced by entry into a nursing home.

**Objective:**

The aim was to discuss the results of previous routine data analyses on the treatment of psoriasis patients in nursing homes with medical providers (dermatologists, general practitioners, nursing service managers, and nursing staff) on the basis of interviews and focus groups, to identify difficulties in the treatment and finally to derive recommendations for action regarding sustainable gerontodermatological treatment.

**Materials and methods:**

Qualitative guideline-based interviews and focus groups with dermatologists (*n* = 5), general practitioners (GPs, *n* = 7), nurses (*n* = 7), and care managers (*n* = 2) were conducted. The data were evaluated by content analysis.

**Results:**

The analyses resulted in 344 statements, which could be categorized into 14 main topics. The results show that there is a need for improvement in the quality of care for people with skin diseases, especially psoriasis, in nursing homes. This is evident at both the medical and nursing levels. According to the providers (*N* = 21), this can be addressed in particular through increased remote consultations (telemedicine), dermatological training for GPs and nurses, and closer cooperation between the individual disciplines.

**Conclusion:**

Remote consultations, but also a specific guideline on the “skin of the ageing person” can be useful to improve the dermatological care situation in nursing homes and thus increase the well-being of those affected.

Über die dermatologische Versorgung von Menschen mit Psoriasis bei Pflegeheimeintritt ist unzureichend bekannt. Auf Basis einer vorausgehenden Sekundärdatenanalyse sollen unter Einbeziehung versorgungsrelevanter Akteure Schwierigkeiten der Versorgung aufgedeckt und diskutiert werden und Handlungsempfehlungen für eine zukunftsfähige gerontodermatologische Versorgung abgeleitet werden.

## Hintergrund

Demografische Veränderungen bewirken einen steilen Anstieg der Anzahl der über 65-Jährigen bei gleichzeitigem Rückgang der Bevölkerung in Deutschland im erwerbsfähigen Alter. Damit verbunden ist der Anstieg der Anzahl pflegebedürftiger, multimorbid Erkrankter und der daraus resultierenden Multimedikation [1]. Derzeit werden in Deutschland rund 800.000 Pflegebedürftige in Pflegeheimen versorgt. Laut aktuellen Vorausberechnungen werden bis zum Jahr 2060 4,5 Mio. Menschen in Deutschland pflegebedürftig und damit 221.000 mehr Menschen betroffen sein als in früheren Projektionen angenommen [2]. Die ärztliche Versorgung von Heimbewohnern erfolgt derzeit durch ambulant tätige Haus- und Fachärzte. Untersuchungen zeigen, dass nahezu alle Bewohner regelmäßigen Kontakt zu Hausärzten haben; andere Facharztgruppen hingegen nur in geringem Maß an der Versorgung beteiligt sind [3, 4]. Nach unserem Kenntnisstand gibt es keine bevölkerungsbezogenen Daten zur dermatologischen Versorgung älterer Menschen in Pflegeheimen [5, 6]. National wie auch international gibt es insbesondere keine Information zur Versorgung von Psoriasiserkrankten im Setting Pflegeheim und zur Frage, wie diese durch den Eintritt in ein Pflegeheim beeinflusst wird. Erste Arbeiten aus Deutschland zur Gerontodermatologie zeigen, dass sich Pflegekräfte in Heimen – als enge Kontaktpersonen der Bewohner im pflegerischen Alltag – im Umgang mit dermatologischen Krankheiten wie auch mit der Nutzung dermatologischer Fachtermini und Hautstatusbeschreibungen unsicher fühlen. Zudem sind Defizite in der Kooperation mit Haus- und Hautärzten zu erkennen [7]. Zusätzlich weisen erste internationale Untersuchungen aus dem Setting Pflegeheim auf, dass der Bedarf an dermatologischer Versorgung in Pflegeheimen hoch ist [8, 9]. Trotz benannter hoher Relevanz, geriatrische Patienten adäquat zu versorgen, haben nur wenige Patienten Kontakt zu Dermatologen [10]. Der jährliche Versorgungsbedarf für dermatologische Erkrankungen wurde in einer populationsbezogenen deutschen Studie bereits unter den 61- bis 70-Jährigen mit fast 40 % angegeben [11]. Es gibt bisher keine Informationen zur Versorgungssituation von Menschen im höheren Lebensalter mit Psoriasis im Kontext einer Pflegeheimaufnahme in Deutschland.

Das durch den Innovationsfonds geförderte Projekt *PsoGero* hat zum Ziel, Informationen darüber zu gewinnen, wie ältere Menschen mit Psoriasis vor und nach Eintritt in ein Pflegeheim versorgt werden. Das Projekt besteht aus zwei Teilbereichen: Im ersten Studienabschnitt wurden anhand von Routinedaten der DAK-Gesundheit (DAK-G) die fachärztliche Versorgung wie auch die Versorgung mit Arzneimitteln im Jahr vor bzw. nach Heimeintritt untersucht. Insgesamt zeigen die Routinedatenanalysen, dass zum einen die Diagnosestellung überwiegend ambulant stattfindet und für die Altersgruppe kaum mittelschwere/schwere Fälle diagnostiziert werden. Hinsichtlich der Komorbidität zeichnen sich eine Zunahme von Demenzerkrankungen und typischen Pflegeproblemen (Dekubitus, Inkontinenz) und eine Abnahme von Depression und Hypertonie ab. Die Daten zeigen, dass alle Bewohner hausärztlich versorgt werden und die Versorgung durch Dermatologen und Internisten abnimmt (besonders deutlich in der höchsten Altersklasse > 85 Jahre), während die neurologische Versorgung zunimmt. Die Diagnose *Psoriasis* wird überwiegend durch den Hausarzt gestellt, lediglich 30 % durch Dermatologen, wobei beide Arztgruppen die Diagnose auch gleichzeitig stellen können. Insgesamt liegt eine Reduktion der Diagnosestellungen durch alle Fachärzte vor. Der wichtigste Prädiktor dafür, auch im Heim dermatologisch versorgt zu werden, ist ein bereits bestehender dermatologischer Kontakt vor Heimantritt. Auch topische Steroide zeigten sich in den Analysen als signifikanter Prädiktor. Außerdem scheinen systemische Therapien (nichtbiologische und biologische) bei der Zielgruppe nicht mehr relevant zu sein (Manuskript eingereicht).

Ziel des zweiten Projektteils war es, anhand von Interviews bzw. Fokusgruppen die Ergebnisse der Routinedatenanalyse mit medizinischen Versorgern (Dermatologen, Allgemeinmedizinern, Pflegedienstleitungen und Pflegekräften) zu diskutieren, mögliche Schwächen aufzuzeigen und abschließend Handlungsempfehlungen für eine zukunftsfähige gerontodermatologische Versorgung abzuleiten.

## Methodik

Die Auswahl der Probanden erfolgte im Sinne eines ‚purposeful sampling‘, d. h. die Stichprobe wurde so ausgewählt, dass sie voraussichtlich im Hinblick auf die Fragestellung möglichst reichhaltige Informationen liefern kann [12]. Für die leitfadengestützten, semistandardisierten Einzelinterviews mit medizinischen Experten sollten je *n* = 3 Personen aus der Versorgung eingeschlossen werden: Dermatologen, Allgemeinmediziner sowie Pflegekräfte und Pflegedienstleitungen (PDL). Für die Fokusgruppen war eine Anzahl von fünf bis acht Teilnehmern (maximal zwei Personen je Expertengruppe) vorgesehen. Die Experten jeder Untergruppe sollten Erfahrung in der Behandlung von Patienten mit Psoriasis haben; als nachrangiges Auswahlkriterium sollte die Heterogenität hinsichtlich Geschlecht, Alter und Berufserfahrung maximiert werden. Das Studienkonzept sah vor, dass die Akquise des ärztlichen Personals über die Kontakte und Vernetzung der Institutsambulanz des Universitätsklinikum Hamburg-Eppendorf (UKE) und der bundesweiten Psoriasisnetze (PsoNet) erfolgt. Die Akquise der anderen Teilnehmer erfolgte über das Netzwerk der Hamburger Pflegegesellschaft e. V. über direkte Kontakte des Instituts für Versorgungsforschung in der Dermatologie und bei Pflegeberufen (IVDP) des UKE und des PsoNet-Vereins, die direkte Ansprache von Pflegeheimen und Aufrufe zur Studienteilnahme in diversen Newslettern und Pflegezeitschriften. Der Beginn der Teilnehmerakquise war für den Zeitraum von Juli bis September 2020 geplant und wurde aufgrund der COVID-19-Pandemie bis Ende Januar 2021 verlängert.

Die Gesamtstichprobe sollte so groß sein, dass die theoretische Sättigung erreicht ist. In Rücksprache mit der Ethikkommission der Ärztekammer Hamburg bestand keine Beratungszuständigkeit der Ethikkommission, da keine Daten verwendet wurden, die sich einem bestimmten Menschen zuordnen lassen. Ebenso erfolgte keine Datenerhebung mit Bezug auf einzelne/individualisierbare Pflegefälle. Die auf Tonträger aufgezeichneten Interviews wurden in anonymisierter Form verschriftlicht und die Aufnahmen gelöscht. Alle Teilnehmer wurden vorab auf die Freiwilligkeit ihrer Teilnahme sowie Datenschutzbestimmungen hingewiesen.

### Datenerhebung

Für die Durchführung der Interviews bzw. Fokusgruppen wurden vorab auf Basis der Ergebnisse der Routinedatenanalyse getrennt Leitfäden für Ärzte, Pflegekräfte und PDLs entwickelt. Die Fragen des Leitfadens bezogen sich u. a. auf Komorbidität, ärztliche Versorgung, Diagnosestellung, Prädiktoren der dermatologischen Versorgung im Pflegeheim, Arzneimitteltherapie und Möglichkeiten der Verbesserung der Versorgung. Die Experten wurden zunächst in Einzelinterviews befragt in der Annahme, dass in einer Gruppenbefragung zusammen mit Kollegen anderer Berufsgruppen über manche Aspekte weniger offen gesprochen werden kann (z. B. Versäumnisse von Ärzten oder Pflegekräften). Aufbauend auf den so gewonnenen Erkenntnissen zur Versorgungssituation von Heimbewohnern mit Psoriasis wurden erste Ideen bezüglich möglicher Interventionsmaßnahmen formuliert und in einen Moderationsleitfaden für die Fokusgruppen überführt, welcher die wichtigsten Fragestellungen beinhaltete. Im Rahmen der Fokusgruppen wurden die so gewonnenen Erkenntnisse und Ideen für Interventionsmaßnahmen diskutiert und weiterentwickelt. Die Interviews/Fokusgruppen wurden von jeweils einer geschulten Moderatorin durchgeführt und dauerten zwischen 15 und 30 min, die Fokusgruppen zwischen 60 und 90 min.

### Auswertung

Alle Interviews und Fokusgruppen wurden digital aufgenommen, wörtlich transkribiert und in die qualitative Analysesoftware NVivo 11 for Windows (QSR International, Melbourne, Australia) importiert. Im Zentrum der computergestützten Auswertung stand die Entwicklung eines Kategoriensystems. Dieses besteht aus Haupt- und Unterkategorien, die primär aus den Themenblöcken der Leitfäden abgeleitet und induktiv durch weitere relevante, im Rahmen der Interviews geäußerten Themen ergänzt wurden. Jede Kategorie wurde durch Definitionen und Ankerbeispiele beschrieben. Das Interviewmaterial wurde darauffolgend im Hinblick auf die deduktiv und induktiv festgelegten Kategorien kodiert und ausgewertet. Jeder Auswertungsschritt wurde dokumentiert, um die Nachvollziehbarkeit der Analyse zu gewährleisten [12]. Um die Reliabilität der Analyse zu gewährleisten, wurde die Analyse von zwei Wissenschaftlern (RS, CCvS) durchgeführt [12]. Alle Interviews und Fokusgruppen wurden nach Auswertung durch beide Wissenschaftler konsentiert.

## Ergebnisse

Von Oktober 2020 bis Januar 2021 wurden insgesamt zwölf Einzelinterviews (je drei Dermatologen und Allgemeinmediziner, fünf Pflegekräfte, eine Pflegedienstleitung) und zwei Fokusgruppen mit je einem Dermatologen, einem Allgemeinmediziner und einer Pflegekraft geführt. In einer der Fokusgruppen nahm zusätzlich noch eine Pflegedienstleitung teil. Die Mehrheit der befragten Experten ist in der Metropolregion Hamburg tätig. Die teilnehmenden Dermatologen und Allgemeinmediziner sind alle in niedergelassener Tätigkeit und seit mehreren Jahren regelmäßig in die Betreuung von gerontodermatologischen Patienten in Pflegeheimen eingebunden. Die befragten Pflegekräfte und Pflegedienstleitungen stammen aus insgesamt sechs verschiedenen Pflegeeinrichtungen, welche sich ebenfalls alle in der Metropolregion Hamburg befinden.

Die Auswertung der Einzelinterviews ergab insgesamt 258 Aussagen, die acht Hauptkategorien mit jeweiligen Subkategorien zugeordnet werden konnten (Tab. 1). Die meisten Aussagen (*n* = 65) wurden der Hauptkategorie ‚Möglichkeiten zur Verbesserung der Versorgung‘ zugeordnet und thematisieren insbesondere den Ausbau der digitalen Versorgung, eine engere Kooperation zwischen den Fachärzten sowie dermatologische Schulungsprogramme für Pflegekräfte und Hausärzte. Alle Teilnehmer befürworteten die zuvor genannten Themen, benannten jedoch auch hiermit verbundene Herausforderungen und Limitationen, wie beispielsweise Grenzen der digitalen Versorgung im Hinblick auf Datenschutz und Qualität der verfügbaren Bilder bzw. Aufnahmen. So berichtete beispielsweise ein Dermatologe: „Ich denke, Telemedizin wird gut sein für die Therapiekontrolle […], aber für die Erstdiagnose-Stellung ist es nicht geeignet.“ Auch Unsicherheiten seitens der Patienten wurden hier als Herausforderungen genannt: „Viele Hauterkrankungen treten ja auch fernab von Gesicht und Händen auf. Und das stelle ich mir für eine alte Dame schon schwierig vor, wenn die sich vor so einer Kamera entblößen soll und dann ihre Hautpartien in eine Kamera hält, die der Hautarzt dann ja auch nur bedingt gut beurteilen kann“ (PDL).

**Table 1:** 

Hauptkategorie	Subkategorie	*N* (%)
Arzneimitteltherapie: systemische Therapien spielen keine Rolle mehr	Systemische Therapien spielen keine Rolle	8 (3,1)
	Grund: Psoriasisaktivität nimmt mit Alter ab – *nein*	5 (1,9)
	Grund: Psoriasis ist nicht therapierelevant – *ja*	13 (5,0)
	Grund: Psoriasis ist nicht therapierelevant – *nein*	2 (0,8)
	Grund: Psoriasisaktivität nimmt mit Alter ab – *ja*	4 (1,6)
Ärztliche Versorgung	Abnahme Versorgung durch Dermatologen und Internisten	5 (1,9)
	Abnahme Versorgung durch Dermatologen und Internisten *– Gründe*	18 (7,0)
	Dermatologische Versorgung ausreichend – *ja*	3 (1,2)
	Dermatologische Versorgung ausreichend – *Gründe ja*	3 (1,2)
	Dermatologische Versorgung ausreichend – *nein*	3 (1,2)
	Dermatologische Versorgung ausreichend – *Gründe nein*	8 (3,1)
	Primär hausärztlich	12 (4,7)
	Zunahme neurologische Versorgung	7 (2,7)
Diagnosestellung	Ambulant	9 (3,5)
	Ambulant – *Gründe*	6 (2,3)
	Idealfall	11 (4,3)
	Primär Hausarzt	10 (3,9)
	Reduktion der Diagnosen durch alle Fachärzte	2 (0,8)
Komorbiditäten	Allgemein	1 (0,4)
	Abnahme Depression, Hypertonie	3 (1,2)
	Keine Zustimmung Demenz – Pflegeproblemehypothese	0
	Keine Zustimmung zu Depressions- und Hypertoniehypothese	4 (1,6)
	Zunahme Demenz, Pflegeprobleme	6 (2,3)
Möglichkeiten zur Verbesserung der Versorgung	Digitale Versorgung – *ja*	13 (5,0)
	Digitale Versorgung – *nein*	3 (1,2)
	Engere Kooperation Fachärzte	14 (5,4)
	Schulungsprogramme für Pflegekräfte	14 (5,4)
	Sonstiges	21 (8,1)
Prädiktoren für Derma-Versorgung im Heim	Bestehende Dermatologieversorgung – Zustimmung Prädiktor	11 (4,3)
	Bestehende Dermatologieversorgung – kein Prädiktor	1 (0,4)
	Nachteile bestehender Dermatologieversorgung	2 (0,8)
	Topische Steroide	9 (3,5)
Schweregrad: Routinedaten haben gezeigt, dass für die Altersgruppe überwiegend milde und kaum mittelschwere/schwere Fälle diagnostiziert werden	Mild – Zustimmung	4 (1,6)
	Mild – Gründe	1 (0,4)
	Moderat, schwer – Zustimmung	6 (2,3)
Unterversorgung Psoriasis	*Ja*	11 (4,3)
	*Ja* – *Gründe*	4 (1,6)
	*Nein*	1 (0,4)
Gesamt	–	258 (100)

*N* Anzahl Kodierungen, *%* prozentualer Anteil an Kodierungen insgesamt

Insgesamt wurden 59 Aussagen der Hauptkategorie ‚ärztliche Versorgung‘ zugeordnet, wobei etwa die Hälfte der Befragten (*n* = 5) eine Abnahme der dermatologischen Versorgung bestätigt und eine damit einhergehende unzureichende Versorgung durch entsprechende Fachärzte. Alle Teilnehmer bestätigten hingegen, dass Betroffene primär hausärztlich versorgt werden.

Der Hauptkategorie ‚Diagnosestellung‘ wurden insgesamt 38 Aussagen zugeordnet. Dabei berichtete die Mehrheit der Befragten (*n* = 9), dass die Diagnose *Psoriasis* überwiegend ambulant und im hausärztlichen Kontext gestellt wird. Auffällig ist hier die unterschiedliche Einschätzung zwischen Dermatologen und Allgemeinmedizinern. Während Allgemeinmediziner der Meinung sind, Psoriasis gut diagnostizieren zu können: „Ja, Psoriasis ist etwas, was Hausärzte gut diagnostizieren können.“, sehen Dermatologen die Diagnose durch Hausärzte eher kritisch: „Ich glaube, viele Hausärzte sind mit der Diagnosestellung einfach überfordert.“.

Die Auswertung der Fokusgruppen ergab insgesamt 86 Aussagen, die sechs Hauptkategorien mit jeweiligen Subkategorien zugeordnet werden konnten (Tab. 2). Die meisten Aussagen konnten der Hauptkategorie ‚Handlungsempfehlungen‘ zugeordnet werden und thematisieren – ähnlich wie bei den Einzelinterviews – neben der digitalen Versorgung, dermatologischen Schulungen und einer engeren Kooperation auch organisatorisch-strukturelle Lösungsansätze, aber auch Herausforderungen und Limitationen. Ungeachtet der jeweiligen Disziplin sprachen die Teilnehmer sich einheitlich für eine verstärkte digitale Versorgung aus: „Man kann sich auch vorstellen, dass in Zukunft teledermatologische Lösungen noch mehr zum Zuge kommen. Dass der Hausarzt im Prinzip der Mittler ist und dann sagt, da muss jetzt nochmal ein Dermatologe draufgucken und der schaltet sich quasi aus seiner Praxis teledermatologisch ins Heim.“ (Allgemeinmediziner). Einig waren sich die Teilnehmer auch in puncto Datenschutz, der aktuell nicht ausreichend gewährleistet wird, sowie noch nicht flächendeckend ausgereifter und verfügbarer technischer Voraussetzungen wie beispielsweise hochwertiger Internetverbindungen. Insgesamt wurden 21 Aussagen der Hauptkategorie ‚Unterversorgung Psoriasis‘ zugeordnet. Ob eine Unterversorgung nach Einschätzung der medizinischen Versorger vorliegt, scheint lokal bzw. je nach Pflegeeinrichtung sehr unterschiedlich zu sein. So berichtet eine PDL: „[…] da einen Dermatologen zu finden […] das ist sehr, sehr schwierig. Wir haben tatsächlich keinen Hautarzt, der bei uns regelmäßig ins Heim kommt. Es wäre wirklich wünschenswert. Viele Kontaktaufnahmen sind da leider auch schon gescheitert.“ Eine Pflegekraft aus einer anderen Einrichtung hingegen äußert sich gegenteilig: „Ich kann das überhaupt nicht bestätigen, weil wir echt dermatologisch gut versorgt sind in dem Heim, wo ich gerade arbeite. Ich kenne diese Problematik einfach gar nicht, dass die Leute, die Psoriasis haben, ihre Visite nicht erhalten oder andere dermatologische Geschichten nicht aufgeklärt werden können.“ Ähnlich heterogen sind die Erfahrungen hinsichtlich der Qualität der dermatologischen Versorgung. Angesichts der Arzneimitteltherapie berichtet die Mehrheit der Befragten, dass ihrer Erfahrung nach systemische Therapien bei der Zielgruppe keine Rolle mehr spielen. So äußerte sich ein Allgemeinmediziner: „Ich habe eher den Eindruck, dass es umgekehrt war, dass medikamentöse Therapien ganz häufig abgesetzt werden, nach dem Motto: Da sind so viele Medikamente, und wir brauchen eine Straffung der Medikation und da fällt das über, was nichts mit Herz und Kreislauf zu tun hat und mit Leber […].“

**Table 2:** 

Hauptkategorie	Subkategorie	*N* (%)
Arzneimitteltherapie: systemische Therapien spielen keine Rolle mehr	Systemische Therapien spielen keine Rolle	5 (5,8)
	Grund: Psoriasisaktivität nimmt mit Alter ab – *nein*	3 (3,5)
	Grund: Psoriasisaktivität nimmt mit Alter ab – *ja*	1 (1,2)
Handlungsempfehlungen	Probleme und Herausforderungen	13 (15,1)
	Organisatorisch, strukturell	4 (4,7)
	Ideale disziplinübergreifende Kooperation	6 (7,0)
	Engere Kooperation Fachärzte	4 (4,7)
	Digitale Versorgung	8 (9,3)
	Dermatologische Schulungen	4 (4,7)
Schweregrad: Routinedaten haben gezeigt, dass für die Altersgruppe überwiegend milde und kaum mittelschwere/schwere Fälle diagnostiziert werden	Kaum schwere Fälle – *ja*	4 (4,7)
	Kaum schwere Fälle – *nein*	1 (1,2)
Keine Therapierelevanz	*Ja*	6 (7,0)
	*Nein*	1 (1,2)
Qualität der Derma-Versorgung	*Ausreichend/gut*	3 (3,5)
	*Verbesserungsbedürftig*	2 (2,3)
Unterversorgung Psoriasis	*Ja*	13 (15,1)
	*Nein*	8 (9,3)
Gesamt	–	86 (100)

*N* Anzahl Kodierungen, *%* prozentualer Anteil an Kodierungen insgesamt

## Diskussion

Dies ist unseres Wissens die erste Studie, die mittels eines qualitativen Ansatzes die Versorgungssituation von älteren Menschen mit Psoriasis vor und nach Eintritt in ein Pflegeheim untersucht. Die Qualität der Versorgung von Menschen in Pflegeeinrichtungen wird vielfach kritisch diskutiert. Dies betrifft auch die Verfügbarkeit fachärztlicher Konsultationen wie auch z. B. die Verordnungen von Psychopharmaka [4]. Die Haut von Menschen des höheren Lebensalters – und dies gilt im Besonderen für Pflegeheimbewohner – wird stark beansprucht, beispielsweise durch Inkontinenz, Immobilität oder auch kognitive Einschränkungen und der damit verbundenen mangelnden Formulierung von Hautzustandsveränderungen [13, 14]. Aktuelle Forschungsaktivitäten widmen sich zunehmend der Hautgesundheit von Menschen im Pflegeheim. Die Versorgungssituation von Menschen mit Psoriasis in Pflegeheimen ist allerdings unerforscht, nicht zuletzt da sie nicht als eine typische Erkrankung des höheren Lebensalters gilt. In Therapieempfehlungen wird diese Personengruppe daher nicht ausreichend berücksichtigt. Im Kontext des demografischen Wandels und der Chronizität der Erkrankung gewinnt sie aber zunehmend an Relevanz in der alltäglichen Gesundheitsversorgung.

Die Ergebnisse zeigen, ähnlich wie die vorausgehenden Routinedatenanalysen auf Basis der DAK‑G, dass für die Versorgungsqualität von Menschen mit Hautkrankheiten in Pflegeinrichtungen ein Verbesserungsbedarf besteht. Dieser zeigt sich sowohl auf ärztlicher als auch auf pflegerischer Ebene. Auch vor dem Hintergrund, dass weitreichende Untersuchungen belegen, dass über 80 % der Menschen im Alter von über 80 Jahren mindestens 1‑mal jährlich Bedarf nach einer dermatologischen Versorgung aufweisen, lassen sich folgende Empfehlungen zur Verbesserung der Qualitätsstandards in der Versorgung von Menschen mit chronischen Hautkrankheiten in Pflegeheimen aus den Ergebnissen ableiten.

### Interventionelle Maßnahmen

Auf ärztlicher Ebene besteht erkennbar eine Verzögerung im Zugang zu Fachärzten, teilweise auch Hausärzten. Es fehlt oftmals die Verfügbarkeit zur rechten Zeit am rechten Ort. Dafür gibt es erste Lösungsansätze, welche teilweise bereits in der Praxis angewendet werden [15, 16]. Vorausgehende Studien haben am Beispiel der Versorgung von Menschen mit chronischen Wunden in Pflegeeinrichtungen bereits gezeigt, dass ein erheblicher Zugewinn und Nutzen in der Einführung telemedizinischer Unterstützung der vor Ort versorgenden Ärzte besteht [15, 16]. Zum Teil können diese mit Konsultationen unterstützt werden, zum Teil sind auch direkte Interventionen auf telemedizinischem Wege in den Pflegeheimen durch geschulte Dermatologen mit telemedizinischer Ausbildung sinnvoll.

In Analogie zur medizinischen Versorgung bedarf auch die Pflegeversorgung durch Personen in der Kranken- oder Altenpflege einer kontinuierlichen Unterstützung, da hier fehlendes Fachwissen aus dermatologischer Sicht vermittelt werden muss. Nach eigenen Berichten aus Heimen ist nur selten in einem Pflegeheim eine Pflegefachkraft als Experte für den Bereich Dermatologie fortgebildet. Auch hier hat sich die Durchführung von Pflegekonsilen auf digitalem Wege im Sinne der Telepflege als wirksam und nutzbringend erwiesen [15, 16]. Auf diese Weise können Pflegeprobleme und -defizite am Patienten frühzeitig erkannt werden, insbesondere im Zuge regelmäßiger Online-Pflegevisiten am Patienten.

Zugleich ermöglicht die Pflegevisite, welche das Ziel hat, über das Befinden und die Entwicklung des Patienten Klarheit zu verschaffen, eine bessere Schulung und ein früheres Feedback-System für die Pflegekräfte, sodass deren Kenntnisstände wie auch die Motivation verbessert werden können [17].

## Perspektivische Maßnahmen zur strukturellen Verbesserung

In der ärztlichen Versorgung von Personen in Pflegeheimen könnte mit Blick auf das beobachtete Defizit in der dermatologischen Versorgung eine spezifische Leitlinie zur „Haut des alternden Menschen“ von Nutzen sein. Eine entsprechende S2-Leitlinie kann zeitlich wie auch inhaltlich umgehend umgesetzt werden. Ein Aufruf an die Arbeitsgemeinschaft der Wissenschaftlichen Medizinischen Fachgesellschaften (AWMF) über die Qualitätskommission der Deutschen Dermatologischen Gesellschaft ist sinnvoll. Des Weiteren ist eine bessere Implementierung dermatologischer Expertise in die Ausbildung von Hausärzten und geriatrisch tätigen Ärzten aller Richtungen zu empfehlen. Die zum Teil Jahre oder Jahrzehnte lang zurückliegende dermatologische Grundbildung vieler Ärzte hält mit dem aktuellen Entwicklungsstand der Dermatologie, insbesondere der Entwicklung moderner therapeutischer Ansätze, nicht mehr mit. Als bestehende Basislektüre für die versorgenden Ärzte in Pflegeheimen sind insbesondere die folgenden Leitlinien zu empfehlen: Konsensus Xerosis cutis [18], Leitlinie topische Therapie der Psoriasis [19], Lehrbuch chronische Wunden [20].

Auf struktureller Ebene ist es wünschenswert, wenn neue Erkenntnisse zur Pflege und Versorgung von Hautkrankheiten auch in die nationalen Pflegestandards einfließen. Zur Disseminierung der Leitlinienempfehlungen aus dem ärztlichen Bereich bieten sich Online-Portale für die Pflege sowie kurze digitale Schulungen an. Diese könnten beispielsweise über die neu entstandenen Pflegekammern organisiert und geleitet werden. Auch die Erarbeitung von leitlinienbasierten Pocket-Versionen für die Kitteltasche wäre eine praktische Möglichkeit, Grundlagen der Hautgesundheit im hohen Lebensalter einerseits, aber auch neueste Erkenntnisse zur dermatologischen Pflege und Versorgung andererseits schnell und praxisnah in den klinischen Alltag einfließen lassen zu können.

## Stärken und Schwächen

Die vorliegende Studie liefert erstmalig Informationen über die Versorgung der vulnerablen Gruppe von Pflegeheimbewohnern mit Psoriasis. Eine wesentliche Limitation der Studie ist die geringe Fallzahl der befragten Experten. Darüber hinaus ist die Mehrheit der befragten Versorger in Norddeutschland/Hamburg tätig. Die Übertragbarkeit der Ergebnisse auf andere Bundesländer, insbesondere ländlichere Regionen bleibt daher begrenzt. Die Möglichkeiten für eine fachärztliche dermatologische Vorstellung von Menschen im Pflegeheim sind in ländlichen Regionen vermutlich noch begrenzter als in Großstädten. Pflegeeinrichtungen in ländlichen Regionen könnten daher umso stärker von digitalen Versorgungsangeboten profitieren. Dies sollte in weiteren Studien Berücksichtigung finden. Eine weitere Limitation der Studie ist, dass sie nicht vollständig die Perspektive der Betroffenen bzw. ihrer Angehörigen mit einbezieht. Lediglich in den Einzelinterviews wurde die Angehörigensicht durch erfahrene Pflegefachkräfte teilweise wiedergegeben und in den Fokusgruppen auch nach der Einschätzung der Betroffenenperspektive gefragt. Der Vorteil jedoch liegt in der Erfassung der Sicht aller an der medizinisch-pflegerischen Versorgung Beteiligten, die für das Gesamtverständnis der Versorgungssituation unabdingbar sind.

Die Ergebnisse sollten daher bei der Umsetzung der beschriebenen Maßnahmen Berücksichtigung finden, um langfristig die Versorgungssituation Betroffener zu verbessern und somit letztlich ihr Wohlbefinden und ihre Lebensqualität zu steigern.

## Fazit für die Praxis

- Es besteht ein Verbesserungsbedarf für die Versorgungsqualität von Menschen mit Hautkrankheiten in Pflegeinrichtungen.
- Neben einer verstärkten fachärztlichen Versorgung sehen die Befragten dazu primär einen Bedarf am Ausbau der telemedizinischen Versorgung.
- Letzteres bedarf insbesondere einer Verbesserung der technischen Lösungen.
